# NAT10-mediated RNA acetylation enhances HNRNPUL1 mRNA stability to contribute cervical cancer progression

**DOI:** 10.7150/ijms.83828

**Published:** 2023-06-26

**Authors:** Yingfei Long, Yifei Ren, Qinglv Wei, Youchaou Mobet, Yujiao Liu, Hongyan Zhao, Tao Liu, Lei Cheng, Ping Yi

**Affiliations:** Department of Obstetrics and Gynecology, The Third Affiliated Hospital of Chongqing Medical University, Chongqing, 401120, China.

**Keywords:** N4-acetylcytidine (ac4C), cervical cancer, HNRNPUL1, mRNA stability, NAT10

## Abstract

N4-acetylcytidine (ac4C) is a lately discovered nucleotide modification that has been shown to be closely implicated in cancer. N-acetyltransferase10(NAT10) acts as an enzyme that regulates mRNA acetylation modifications. Currently, the role of NAT10-mediated RNA acetylation modification in cervical cancer remains to be elucidated. On the basis of transcriptome analysis of TCGA and GEO open datasets (GSE52904, GSE29570, GSE122697), NAT10 is upregulated in cervical cancer tissues and correlated with poor prognosis. Knockdown of NAT10 suppressed the cell proliferation, invasion, and migration of cervical cancer cells. The in vivo oncogenic function of NAT10 was also confirmed in xenograft models. Combined RNA-seq and acRIP-seq analysis revealed HNRNPUL1 as the target of NAT10 in cervical cancer. NAT10 positively regulate HNRNPUL1 expression by promoting ac4C modification and stability of HNRNPUL1 mRNA. Furthermore, depletion of HNRNPUL1 suppressed the cell division, invasion, and migration of cervical cancer. HNRNPUL1 overexpression partially restored cellular function in cervical cancer cells with NAT10 knockdown. Thus, this study demonstrates that NAT10 contributes to cervical cancer progression by enhancing HNRNPUL1 mRNA stability via ac4C modification, and NAT10-ac4C-HNRNPUL1 axis might be a potential target for cervical cancer therapy.

## 1. Introduction

Cervical cancer (CC) is the 4th most popular female cancer globally, with the highest frequency and death 1,2. Infection of human papillomavirus (HPV) is the primary trigger of cervical cancer development and malignancy, but not all HPV-infected individuals advance to metastatic cervical cancer. The presence of HPV DNA is insufficient to induce cervical cancer and may require additional genetic and epigenetic events. FIGO staging of cervical cancer is the most widely used staging system for cervical cancer worldwide. However, even within the same FIGO stage, survival rates vary widely, suggesting the need for additional prognostic and predictive markers that reflect the biodiversity of cancer and guide personalized treatment 3. Currently, therapeutic possibilities for cervical cancer imply immunotherapy, chemotherapy, radiation therapy, and surgery. However, the effectiveness of clinical treatment for advanced or recurrent cervical cancer is low, and the prognosis for patients is not good. Therefore, in addition to HPV testing, there is a prompted demand to search for new biomarkers related to the development and prognosis of cervical cancer.

Epigenetics implies genetic alterations in gene transcription and cellular characteristics initiated by the processes without altering the DNA sequence. Epigenetics is tightly linked to major human diseases, including autoimmune diseases, neurodegenerative diseases, and tumors. Genetic studies include DNA methylation, histone modifications, and chromatin remodeling. Among these, RNA epigenetic modification is a research topic that has received much attention in recent years. More than 170 RNA modifications have been discovered to date, enriching the diversity of genetic information and dramatically impacting human physiology and pathology 4,5,6. The major modifications on mRNA discovered to date include 5-methylcytosine (m5C), N6-methyladenosine (m6A), N1-methyladenosine (m1A), pseudouridine (Ψ) and 2'-O-methylation, ribose methylation (Nm). ac4C is an old and well-preserved RNA modification discovered early on rRNA and tRNA and catalyzed by NAT10 and its related proteins 7,8. Arango et al. found numerous ac4C modifications on mRNAs and that ac4C alters mRNA stability and translation efficiency 9. mRNA stability and translation efficiency are involved in many vital developments, such as tumorigenesis and immune response, by regulating gene expression, which is central to biological activity 10,11.

N-acetyltransferase 10 (NAT10) belongs to the GCN5-related N-acetyltransferase (GANT) superfamily and functions as a histone and non-histone acetyltransferase 12,13. NAT10 is the only protein with an acetylase structural domain and an RNA-binding structural domain, and is thought to be an RNA ac4C modifier 9. NAT10 plays multiple roles in cells, including DNA damage repair 14,15,16, fatty acid metabolism, scotides, iron death, microtubule acetylation, cell cycle, and mitosis. NAT10 is abundantly transcribed in various tumor tissues, including pancreatic 17, prostates 18, bladders 19, stomachs 20, colons 21, liver 22 and is involved in the biological processes of cell proliferation, invasion, and metastasis. Thus, NAT10 is a promising novel genetical marker and specific therapeutic biomolecule for the detection of malignancy. Currently, RNA ac4C modification mediated by NAT10 in cervical cancer development is primarily unresolved.

In this work, we revealed that overexpression of NAT10 is coupled with low prognosis in cervical cancer patients and that NAT10 suppresses tumorigenesis in cervical cancer. Multi-omics screening identified HNRNPUL1 as the primary target of NAT10. NAT10 interacts with HNRNPUL1 mRNA to promote HNRNPUL1 expression by enhancing its stability. Furthermore, we identified that expression of HNRNPUL1 is positively associated with expression of NAT10 in cervical cancer and that overexpression of HNRNPUL1 could rescue NAT10 knockdown-mediated tumor inhibition. In conclusion, this work shows the essential role of the NAT10-ac4C-HNRNPUL1 axis in cervical cancer progression.

## 2. Materials and Methods

### 2.1 Cell Culture

Cell lines (SiHa, Hela and HEK293T) were obtained from Cell Line Resource, the National Infrastructure (Beijing, China). MEM (GIBCO, USA) was used for SiHa cells and DMEM (GIBCO, USA) was used for HEK293T and Hela cells. The culture mediums were supplemented with penicillin/streptomycin (1%, GIBCO, USA) and fetal bovine serum (10%, GIBCO, USA). All cell cultures were performed at 37°C in a 5% CO2 atmosphere with 100% humidity.

### 2.2 RNA isolation and qRT-PCR

Whole RNA was purified from cultured cells via TRIzol® (Invitrogen, USA) following the company's instructions. cDNA was synthesized using HiScriptII® 1st Strand cDNA Synthesis Kit (Vazyme, China) following the company's instructions. Target gene expression was normalized using housekeeping genes (GAPDH or 18S rRNA), and the expression of target gene was analyzed by the proportional CT protocol. The sequences for qPCR primers used for detection are indicated in the Supplementary Table.

### 2.3 RNA-seq and bioinformatics analysis

Whole RNA was isolated from control or NAT10-deficient cells via TRIzol® (Invitrogen, USA) following the company's instructions. Library formation were operated by Majorbio (Shanghai, China). RNA sequencing (RNA-seq) was carried out by Majorbio and the expression profile was obtained by analyzing the RNA-seq data with the online Majorbio Cloud Platform (www.majorbio.com). FeatureCounts (version 1.6.3) (http://subread. sourceforge.net) was used to calculate reads mapped to the genome. DESeq2 R-package was run to analyze differences in gene expression. GO annotation analysis was performed using Blast2GO software (version 4.1.9) (https://www.blast2go.com/), which is an automated tool for the assignment of GO terms. RNA-seq read data were visualized with the Integrative Genomics Viewer (version 2.4.8) (https://software.broadinstitute.org/software/igv/). The RNA-seq data has been deposited in the Gene Expression Omnibus database (GSE231762).

### 2.4 Plasmids, cell transfection and lentiviral infection

The pLKO.1 plasmid with NAT10 or HNRNPUL1 short hairpin RNA (shRNA) was constructed to knock down NAT10 or HNRNPUL1 gene expression. jetPRIME transfection reagent (Polyplus, France) was used for the plasmid DNA transfection following the company's instructions. For lentiviral packaging, both plasmid vectors for transgene and packaging, psPAX2 (#12260, Addgene, USA) and pMD2.G (#12259, Addgene, USA) were used for co-transfection into HEK293T cells using jetPRIME transfection. After 48h, viruses were recovered. For generating stable cell lines, lentiviruses with short hairpin RNA (shRNA) were used for transfection into the cells with polybrene (5 ug/mL). 48 hours later, cells were selected with 2 μg/ml puromycin (#ST551, Beyotime, China) for another 48 h. Overexpression vectors containing pcDNA3.1-HNRNPUL1 were generated by inserting the full-length DNA sequence of HNRNPUL1 into the pcDNA3.1 plasmid vector (Youbio, China). Cells were transfected with HNRNPUL1 overexpression vector or empty vector and selected in puromycin. The sequences of the shRNAs are denoted in the Supplementary Table.

### 2.5 Assay for cell proliferation

For the cell proliferation assay, 2500 Hela cells or 3000 SiHa cells corresponding to each group were seeded per well in 96-well plates. Cell numbers were detected using Cell Counting Kit-8 (CCK-8, DOJINDO, Japan) for 5 days. CCK-8 solution (10 uL) was added to the plates and placed at 37°C in 5% CO2 incubator for 2 hours according to the CCK-8 cell growth assay protocol. Optical absorbance was then measured at 450 nm and 630 nm absorbance using a microplate reader (BioTek, USA).

### 2.6 Assay for colony formation

An assay for colony formation was accomplished to quantitate cell proliferative capacity *in vitro*. Briefly, 2000 Hela or 2500 SiHa cells corresponding to each group were resuspended and placed in 6-well plates. After 7-14 days of culture, cells were used for fixation with paraformaldehyde (4%) for 15 min. 0.1% crystal violet (Sigma-Aldrich, USA) was used for cell staining for 30 min. Stained colonies were washed twice and photographed. Colonies consisting of 50 or more cells were evaluated. Triplicate wells were used for each assay.

### 2.7 Transwell migration and invasion assays

For transwell cell migration assays, chambers (Millipore, Germany) were present in a 24-well culture plate. DMEM supplemented with 20% FBS (600 ul) was added to the well's bottom. Cells migrating to the bottom layer were used for fixation with paraformaldehyde (4%) for 20 minutes. Staining with crystal violet (0.1%) was done for approximately 20 minutes. The number of migrated cultured cells was imaged and quantified. For the transwell cell invasion assay, a similar protocol used for the migration assay was followed with the 10% Matrigel (Sigma-Aldrich, USA) pre-coated chamber inserts.

### 2.8 Xenografts in nude mice

Tumor formation was induced by subcutaneous injection of 5 x 10^6^ shNAT10 and shNC Hela cells dissociated in PBS (100 uL) into one side of stably transfected 5 weeks old BALB/c female nude mice. Growing tumors were evaluated every week. Size of tumors were assessed using the following formula: Volume (mm^3^) = (width)^2^ x length/2. After 26 days, animals were terminated and the weight of generated tumors was evaluated. Animal care and research protocols were conducted under a program (2022033) admitted by the Ethics Committee of the Third Affiliated Hospital of Chongqing Medical University.

### 2.9 Western blotting

Alysis solution with protease inhibitors (APExBIO, USA) was used for cell lysis. Protein levels were evaluated with a BCA protein assay kit (Solarbio, China). Cell lysates were used for centrifugation (12,000 g) for 10 min at 4°C to discard cell debris. Proteins were then used for SDS-PAGE and a PVDF membrane was used for protein transfer. Following TBST with 5% nonfat milk, which was used for blocking the membranes, primary antibodies against NAT10 (1:2000, ab251186, Abcam, USA), HNRNPUL1 (1:1000, DF781, Affinity), GAPDH (1:1000, 60004-1-Ig, Proteintech, USA) were used for the incubation of the membranes overnight at 4°C. After 3 times TBST washing (10 min each), a secondary antibody conjugated with horseradish peroxidase (HRP) (1:1000, SA00001-1/SA00001-2, Proteintech, USAds) was used for the membranes for 1 hour on a shaker. After 3 times TBST washing (10 min each), protein bands were evaluated via ECL-Plus reagent (Millipore, USA). ChemiDoc Touch Imaging System (Bio-Rad, USA) was used for signal capture.

### 2.10 RNA immunoprecipitation (RIP) assay

RIP assays were accomplished following the company's instructions using the Magna RIP Kit (Millipore, Germany). Hela and SiHa cells were seeded in petri dishes at approximately 80% confluency. Briefly, magnetic beads coated with 3 ug anti-NAT10 (Abcam, UK) and the corresponding control anti-rabbit IgG (Millipore, Germany) were used for overnight incubation at 4°C for the prepared indicated cell lysate (approximately 2 x 10^7^ cells for each sample). After washing three times with wash buffer containing 150 mM NaCl, HEPES 20 mM, 5 mM MgCl2, 5 mM EDTA, 150 mM KCl, 10% glycerol, and 0.5% NP40, inputs and coprecipitated RNA were eluted from the immunoprecipitated complex, purified, and further evaluated by qPCR. Obtained results were used for the normalization to inputs. RT-qPCR and RNA preparation was accomplished as described above.

### 2.11 Acetylated RNA Immunoprecipitation (acRIP)

Isolation of whole RNA isolation was performed following the instructions described above. acRIP assays were performed following the company's instructions for the Magna MeRIP m6A Kit (Merck Millipore, Germany). shNAT10 and shNC totaled 100 ug of RNA, which was fragmented into 100 to 150 bp lengths. Approximately 10% of the fragmented RNA was then aliquoted as input. The rest of the RNA was used for anti-N4-acetylcytidine (ac4C) antibody (ab252215, Abcam, USA) incubation followed by protein A/G magnetic beads (Invitrogen, USA) at 4°C for 2 h. After several washes, ac4C-rich RNA was purified. The acetylated RNA purified via the above protocol was used for reverse transcriptase reaction and evaluated by qPCR.

### 2.12 RNA stability analysis

24-well plates were used for cell seeding, which was treated with 5 mg/mL actinomycin-D (Act-D, AbMole, USA) for 0, 3, 6, and 9 h. Following incubation for the specified time, cells were collected and mRNA levels were quantified by RT-qPCR to confirm HNRNPUL1 stability. 18S rRNA was utilized for normalization.

### 2.13 Statistical Analysis

Mean ± SD was used for data evaluation, and statistical analysis for comparing the two groups was performed using a two-tailed Student's t-test with GraphPad Prism 8.0. was used for comparison between the two groups. On the other hand, a one-way ANOVA analysis of variance was utilized for differences among the multiple groups. To estimate the relationship between NAT10 and HNRNPUL1 expression levels in cervical cancer, Spearman rank correlation analysis was used to estimate. P values lower than 0.05 were judged statistically significant; *p < 0.05, **p < 0.01, ***p < 0.001, NS, not significant.

## 3. Results

### 3.1. Overexpression of NAT10 in cervical cancer predicts low prognosis

**Figure 1:**
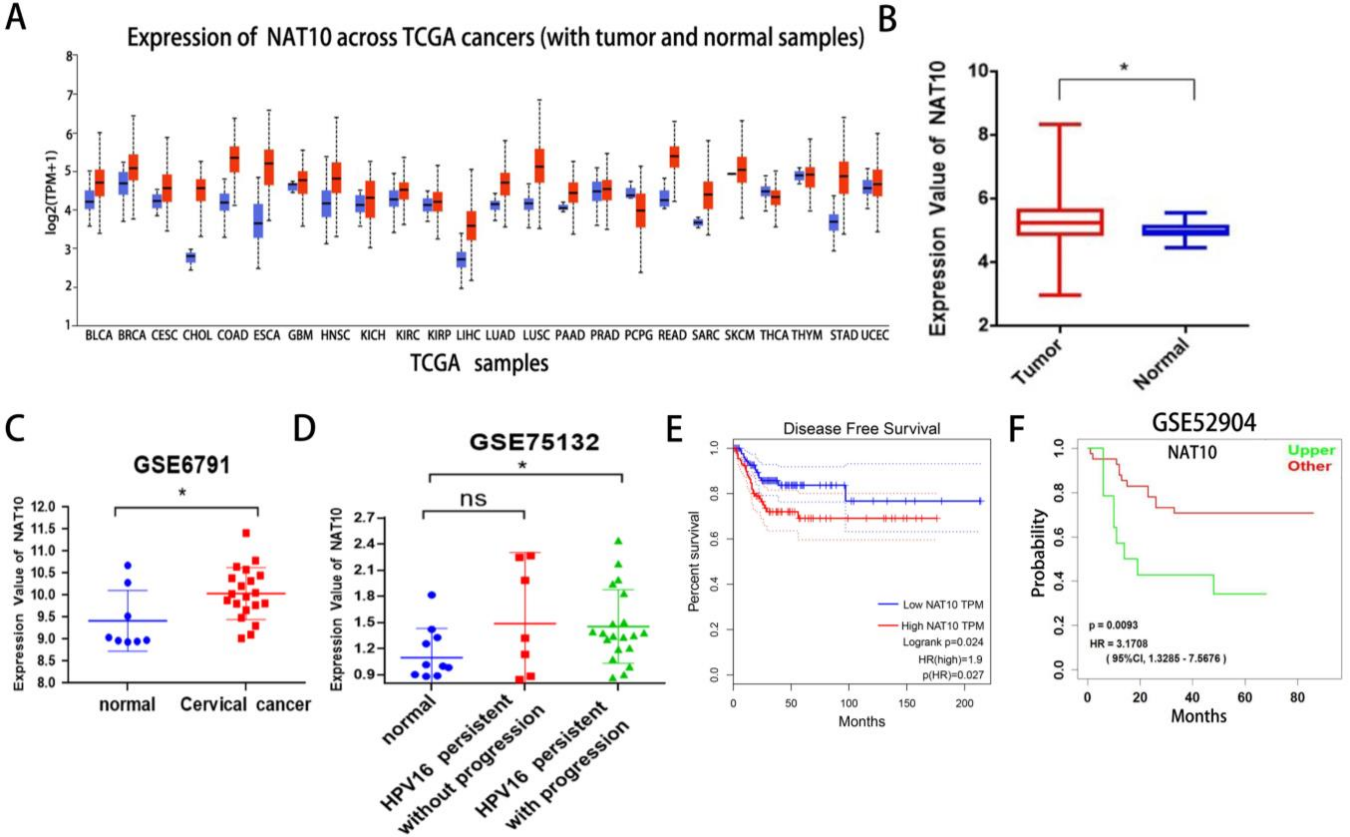
** NAT10 is overexpressed in cervical cancer. (A)** NAT10 expression by the UALCAN platform for pan-cancer analysis. **(B)** Expression of NAT10 in cervical cancer or paired control tissues combined with TCGA and GTEx datasets. **(C-D)** Relative mRNA levels of NAT10 in GEO datasets (GSE6791 and GSE75132). **(E)** Prognostic value of NAT10 in cervical cancer (Disease-free survival in Kaplan-Meier plotter). **(F)** Progression-free survival for NAT10 was performed using LOGpc. Experimental data are indicated as mean ± SD. ANOVA or Student's t test was used for statistical significance analysis. NS, not significant, *P < 0.05, **P < 0.01, ***P < 0.001.

First, we found that NAT10 is differentially expressed in pan-cancer and up-regulated in multiple cancer types in the UCLCAN database (Fig. 1A). Next, we examined NAT10 expression in cervical cancer in the Genotype-Tissue Expression (GTEx) project and the Cancer Genome Atlas (TCGA) dataset (Fig. 1B). Two GEO datasets (GSE6791 and GSE 75132) (Fig. 1C, D), we noticed that NAT10 is significantly up-regulated in cervical cancer compared to normal cervical epithelial tissue. Kaplan-Meier survival analysis from the GEPIA data analysis platform showed that cervical cancer patients with high NAT10 expression had poorer Disease-free survival (DFS) compared to cervical cancer patients with low NAT10 expression, indicating that NAT10 expression was negatively associated with DFS for cervical cancer patients (Fig. 1E). In addition, we analyzed survival probability of the cervical cancer patients with high NAT10 expression (n = 41) and that of low NAT10 expression (n = 14) in the GSE52904 dataset by the LOGpc data platform, and the results demonstrated that high expression of NAT10 was significantly associated with poor prognosis of cervical cancer patients (Fig. 1F), suggesting that NAT10 may be an important marker for the prognosis of cervical cancer patients.

### 3.2. Knockdown of NAT10 inhibits invasion, migration and cell proliferation of cervical cancer

**Figure 2:**
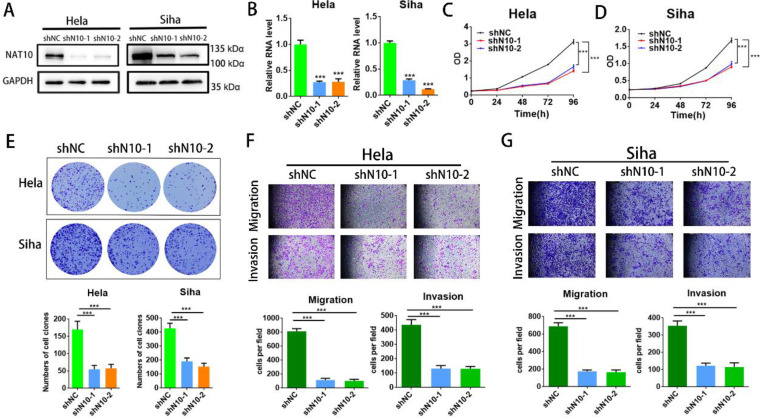
** Knockdown of NAT10 inhibited cervical cancer cell growth and metastasis. (A,B)** Western blotting and RT-qPCR detecting NAT10 knockdown efficiency in Hela and SiHa cells. **(C, D)** Cell growth detected by CCK-8 assay showed that knockdown of NAT10 suppresses cervical cancer cell proliferation. **(E)** Colony formation assay demonstrated that the number of colonies of cervical cancer cells was markedly decreased following NAT10 silencing. **(F, G)** Transwell assays for cell invasion and migration were performed in SiHa and Hela cells by knockdown of NAT10. The scale bar denotes 200 μm. Experimental data are indicated mean ± S.D.

To assess the cellular function of NAT10 in cervical cancer, NAT10 was silenced in cervical cancer cells (SiHa and Hela cells) utilizing two different shRNAs. RT-qPCR analysis and Western blotting indicated that NAT10 was significantly silenced in SiHa and Hela cells (Fig.2A, B), and CCK-8 assays were performed to confirm that NAT10 knockdown effectively reduced cell proliferative capacity in SiHa and Hela cells (Fig. 2C, D). In the colony formation assay, down-regulation of NAT10 markedly decreased the number of colonies in both SiHa and Hela compared to the shNC group (Fig. 2E). In addition, transwell assays were performed on both cells to evaluate the cellular abilities of invasion and migration (Fig. 2F, G). These results suggest that deletion of NAT10 inhibits growth, invasion, and migration of cell lines derived from cervical cancers.

### 3.3. Loss of NAT10 suppresses tumor growth of cervical cancer cells

**Figure 3:**
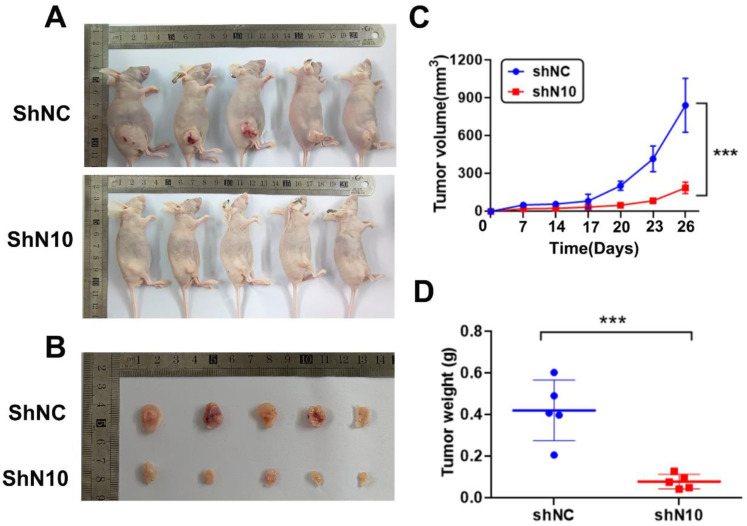
** NAT10 depletion suppresses tumor development of cervical cancer cells in animals. (A)** Nude mice (five-week-old) were used for subcutaneous injection with 1 x10^6^ Hela cells to form subcutaneously implanted tumors. **(B)** The outcomes demonstrated that the knockdown of NAT10 suppresses the development of xenograft tumors of cervical cancer cells. **(C, D)** NAT10 knockdown reduced the weight and volume of tumor formation in nude mice compared with the control group. (n = 5 per group). All values presented were expressed as mean ± SD.

To further explore the NAT10 *in vivo* tumorigenic function, subcutaneous tumorigenesis was performed in nude mice. Hela cells with control or NAT10 knockdown were subcutaneously transplanted into 5-week-old mice. Mice were sacrificed for tumor isolation 26 days after cell transplantation (Fig. 3A). The volume of tumors was markedly reduced in the NAT10 knockdown group compared with the control group (Fig. 3B). Tumor growth curves were generated for the different groups, confirming that knockdown of NAT10 restrained tumor formation in nude mice when compared with controls (Fig. 3C, D). These results demonstrate the cervical cancer-promoting role of NAT10 *in vivo*.

### 3.4. Screening of the candidate target for NAT10 in cervical cancer

**Figure 4:**
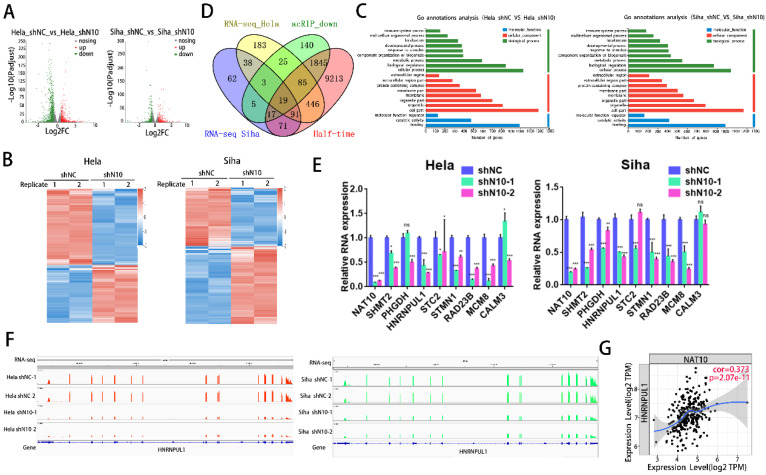
** Identification of candidate targets of NAT10 in cervical cancer. (A)** Volcano plot of RNA-seq dataset of control and knockdown of NAT10 in SiHa and Hela cells. **(B)** Heat map of differentially expressed genes identified by RNA-seq. Red represents genes with high differentially expressing and blue represents genes with low differentially expressing. **(C)** Gene annotation analysis of differentially expressed genes between control and NAT10 knockdown Hela and SiHa cells. **(D)** Venn diagram showing potential targets of NAT10 by combined analysis of RNA-seq, reported acRIP-seq and half-life determination data from this study. **(E)** Validation of candidate target genes by qRT-PCR in SiHa and Hela cells, respectively. **(F)** Sequence information of RNA-seq data in SiHa and Hela cells visualized by Integrative Genomics Viewer (IGV). **(G)** HNRNPUL1 expression was positively associated with NAT10 expression based on the TIMER database.

To investigate the principal mechanism of NAT10 in cervical cancer, RNA-seq was accomplished in SiHa and Hela cells with NAT10 knockdown and in control cells. Volcano plots and heat maps showed the differences in gene expression profiles in SiHa and HeLa cells after knockdown of NAT10, with 736 up-regulated and 970 down-regulated genes in HeLa cells and 449 up-regulated and 337 down-regulated genes in SiHa cells (Fig. 4A, B). GO annotation analysis revealed that these differential genes were mainly enriched in crucial tumor-related signaling pathways and biological processes. These results suggest that NAT10 plays an essential role in the development of cervical cancer (Fig. 4C). Next, we combined the RNA-seq obtained in this study with the reported analysis of acRIP-seq and half-life determination data in Hela cells. The number of dedicated and common genes in each comparison is shown (Fig. 4D). The final multi-omics intersection yielded 19 genes. After literature review and database filtering, a final set of genes of interest (SHMT2, PHGDH, HNRNPUL1, STC2, STM1, RAD23B, MCM8, and CALM3) were selected as candidate target genes. As a result, the regulatory patterns of six genes (SHMT2, PHGDH, HNRNPUL1, STM1, RAD23B, and MCM8) evaluated by RT-qPCR were similar to the results from RNA-seq (Fig. 4E). Previous works have reported that NAT10 is tightly linked to DNA damage 23,24. Therefore, we speculated that NAT10-mediated RNA acetylation modifications might also be involved in the DNA damage process. HNRNPUL1 has been reported to be involved in DNA damage repair^25^,^26^. Therefore, we selected HNRNPUL1 as the target gene of NAT10 in cervical cancer.RNA-Seq reads were then plotted and visualized in the Integrative genomics viewer (IGV) browser. The IGV plot in cervical cancer cells shows changes in the RNA-seq peak of HNRNPUL1. Knockdown of NAT10 reduced the abundance of HNRNPUL1 mRNA in SiHa and Hela cells (Fig. 4F). Analysis of the relationship between HNRNPUL1 and NAT10 expression using the online Tumour Immune Estimation Resource (TIMER) database suggested a tight connection between the gene expression of these two genes (Fig. 4G).

### 3.5. NAT10 regulates the HNRNPUL1 gene expression and RNA stability in cervical cancer

**Figure 5:**
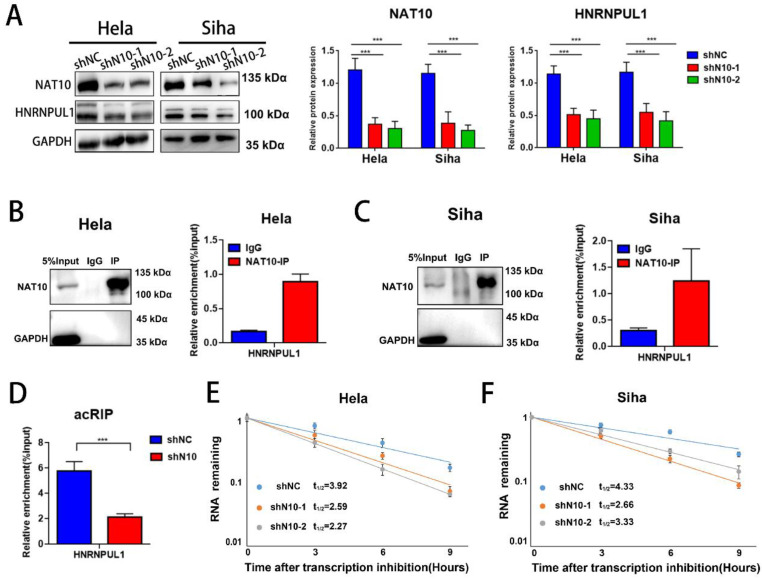
** NAT10 stabilizes HNRNPUL1 mRNA in cervical cancer. (A)** NAT10 and HNRNPUL1 protein levels were identified in cervical cancer cells with NAT10 knockdown. **(B, C)** RIP-PCR assay confirmed the interaction of NAT10 and HNRNPUL1 mRNA in SiHa and Hela cells. IgG was utilized for internal control. **(D)** The acRIP-PCR assay detected ac4C modification of HNRNPUL1 mRNA in Hela cells upon NAT10 knockdown. **(E, F)** RNA stability experiments showed that NAT10 knockdown causes degradation of HNRNPUL1 mRNA. Experimental data are denoted as mean ± S.D.

To decode the regulatory role and mechanism of NAT10 on HNRNPUL1 in cervical cancer, we examined the expression of HNRNPUL1 by NAT10 knockdown by Western blotting and found that NAT10 inhibition significantly reduced HNRNPUL1 protein expression was significantly reduced (Fig. 5A), and RIP-PCR assays in both Hela and SiHa cells confirmed that NAT10 specifically interacts with HNRNPUL1 mRNA (Fig. 5B, C). By meRIP-PCR, HNRNPUL1 mRNA was ac4C modified and was markedly decreased upon knockdown of NAT10 (Fig. 5D). These results indicate that NAT10 may activate HNRNPUL1 expression in an ac4C-dependent manner. Furthermore, RNA stability measurements indicated that NAT10 knockdown promotes HNRNPUL1 mRNA decay (Fig. 5E, F). These findings suggest that HNRNPUL1 is regulated by NAT10-mediated ac4C modification in cervical cancer.

### 3.6. HNRNPUL1 functions as an oncogenic development in cervical cancer

**Figure 6:**
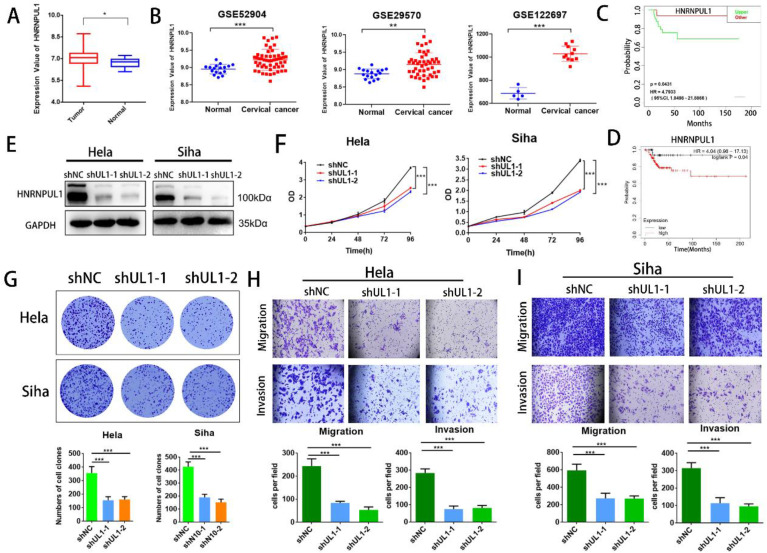
** HNRNPUL1 acts as an oncogene in cervical cancer. (A)** HNRNPUL1 expression levels in cervical cancer and paired control tissues in the TCGA combined with GTEx dataset. **(B)** Expression levels of HNRNPUL1 RNA in cervical cancer tissues and paired control tissues in the GSE52904, GSE29570, and GSE122697 datasets. **(C)** Disease-free interval for HNRNPUL1 was calculated using the online dataset LOGpc with TCGA data. **(D)** Progression-free survival of HNRNPUL1 was evaluated according to the Kaplan-Meier Plotter database. **(E)** Western blotting measuring HNRNPUL1 protein in SiHa and Hela cells transfected with shRNA control (shNC) and shRNA of HNRNPUL1 (sh1 or sh2). **(F)** Cell proliferation of control and HNRNPUL1-deficient SiHa and Hela cells was detected by CCK8 assay. **(G)** The cell proliferation ability was shown by colony formation assay in HNRNPUL1 knockdown and control cell lines. **(H, I)** Cell invasive and migration capacity following transwell chamber assay with knockdown and HNRNPUL1. The scale bar denotes 200 µm. Experimental data are denoted as mean ± S.D.

Next, we analyzed the association between HNRNPUL1 expression in cervical cancer and patient prognosis using public clinical databases. The outcomes demonstrated that expression of HNRNPUL1 was higher in cervical cancer compared to normal cervical epithelial tissue (Fig. 6A, B). Analysis based on TCGA data showed that the group for HNRNPUL1-high had poor Disease-free interval (DFI). (Fig. 6C). Kaplan-Meier Plotter demonstrated that expression of HNRNPUL1 was negatively correlated with Progression-free survival (PFS) in patients with cervical cancer (Fig. 6D). To explore the function of HNRNPUL1 in cervical cancer, silencing of HNRNPUL1 expression in SiHa and Hela cells was verified by Western blotting (Fig. 6E) and CCK-8 assays showed that HNRNPUL1 knockdown significantly suppressed cell growth (Fig. 6F). Furthermore, an analogous trend was detected in the colony formation assay (Fig. 6G), suggesting that HNRNPUL1 knockdown inhibits the abilities of cell growth and colony-forming in cervical cancer cells. Furthermore, transwell experiments demonstrated that silencing HNRNPU1 suppressed the migratory and invasive potential of cervical cancer cells (Fig. 6H, I). These outcomes indicate that HNRNPUL1 is an oncogenic factor during cervical cancer development.

### 3.7. Overexpression of HNRNPUL1 partially rescues the progression of cervical cancer cells that was suppressed by NAT10 silencing

**Figure 7:**
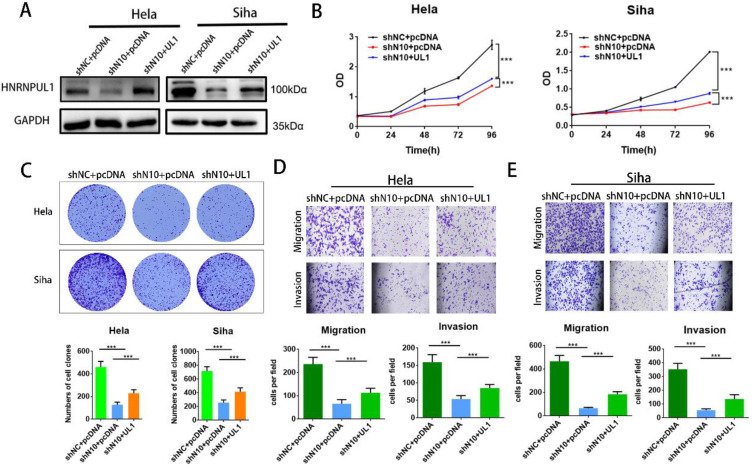
** Overexpression of HNRNPUL1 partially restored the progression of cervical cancer cells inhibited by NAT10 silencing. (A)** HNRNPUL1 protein levels were rescued in NAT10 knockdown cells. **(B)** The CCK-8 assay was used for cell proliferation. **(C)** Overexpression of HNRNPUL1 partially rescued NAT10-induced loss of colony-forming ability. **(D, E)** Cell invasion and migration were measured using transwell assays. The scale bar, 200 um. Experimental data are shown as mean ± S.D.

To further elucidate the involvement of HNRNPUL1 in NAT10-mediated CC progression, rescue experiments were performed: HNRNPUL1 overexpression vectors or control vectors were co-transfected into NAT10-silenced CC cells. Western blotting data demonstrated that expressions of HNRNPUL1 protein were rescued in NAT10 knockdown cells (Fig. 7A). CCK8 and clonogenic assays showed the up-regulation of HNRNPUL1 in SiHa and Hela cells (Fig. 7B, C), indicating that up-regulation of HNRNPUL1 can partially rescue the inhibition of cell growth viability caused by silencing of NAT10 (Fig. 7B, C). In contrast, the knockdown of NAT10 suppressed the migration and invasive potential of cervical cancer cells, but the up-regulation of HNRNPUL1 partially reversed these effects (Fig. 7D, E).

## 4. Discussion

In this work, we found that in cervical cancer, NAT10 is markedly over-expressed and thus is tightly linked with poor prognosis in patients with cervical cancer. NAT10 is essential for cervical cancer cell growth and metastasis. We hypothesized that NAT10 expression could be a potential therapeutic focus for cervical cancer. Combining multi-omics and bioinformatics analyses, we found that NAT10-mediated ac4C acetylation may affect the stability and expression of HNRNPUL1 mRNA in cervical cancer cells. NAT10 is a histone acetyltransferase member of the GNAT family 27,28. It has been reported that NAT10 is a possible clinical target for a variety of progressive diseases, including cancer and Hutchinson-Gilford-Progeria syndrome (HGPS) 29,30. Post-transcriptional modifications of RNA play an essential role in disease pathogenesis. ac4C, as a chemically modified nucleoside, has been implicated in autoimmune diseases 31,32, metabolic diseases 33,34, inflammation 35 and cancer 36,37 and may be involved in a wide range of other diseases. The ac4C on tRNA, rRNA and mRNA are all catalyzed by NAT10 or other homologous enzymes 38. NAT10 can bind to RNA and is the only mRNA acetylation regulator in eukaryotes identified to date that transfers ac4C on mRNA to maintain efficient translation and stabilize mRNA. Current studies suggest that NAT10-mediated RNA ac4C modification is involved in the pathophysiology of various diseases, including viral replication and stability 39,40, spermatogenesis 41, and tumor progression 42,43,44. NAT10 is a virally encoded transcription factor, HIV-1 Tat, and has been shown to bind strongly to Tat and play a central role in viral transcription 45. The ac4C modification can enhance gene expression of HIV-1 by promoting viral RNA stability. Down-regulation of viral structural genes was clearly observed in NAT10 knockdown cells, and luciferase showed similar experimental results 40. The NAT10 inhibitor remodelin could inhibit HIV-1 replication at concentrations without affecting cell viability, indicating that N4-acetylcytidine plays an essential role in infection with viruses and can regulate enterovirus 71 replication and virulence 39. We speculate that in cervical cancer, NAT10-mediated ac4c modification regulates viral virulence by affecting HPV viral replication and stability. This may be another novel mechanism by which NAT10-mediated ac4C modification is involved in tumor progression. Further detailed studies are needed and may offer novel insights into antiviral therapy for cervical cancer.

What is the function of NAT10-mediated RNA ac4C modification in cervical cancer? The purpose of this report was to explore the role and mechanism of NAT10-mediated ac4C modification in cervical cancer. The outcomes indicate that HNRNPUL1 is a direct target of ac4C modification of mRNA by NAT10. Hnrnpul1/early region 1B-associated protein 5 (E1B-AP5) of adenovirus belongs to a heterogeneous nuclear ribonucleoprotein (hnRNP) protein family. It plays essential roles in a wide range of biological processes, including RNA splicing and transcriptional regulation 46. In addition, hnRNPUL1 regulates cellular functions against DNA damage 47. HNRNPUL1 directly associates with p53 and suppresses transcriptional activity of p53 following UV irradiation. In addition, Hong et al. found that HNRNPUL1 down-regulates the expression of PARP1 by controlling gene transcription and increases cellular sensitivity to double-strand breaks (DSBs) 48. Cells silencing HNRNPUL1 were shown to be more sensitive to DNA damage 49. HNRNPUL1 suppresses cisplatin (CDDP) sensitivity of esophageal squamous cell carcinoma cells by regulating the formation of MAN1A2, a cyclic RNA (circRNA) that suppresses CDDP sensitivity 25. HNRNPUL1 protein promotes viral RNA synthesis and Ebola virus infection 50. Therefore, further investigation is warranted to determine whether HNRNPUL1, whose expression is up-regulated by NAT10, may either cause disordered DNA damage repair or promote HPV viral DNA synthesis and infection, thereby exacerbating cervical cancer progression. In conclusion, we show that ac4C modification by NAT10 promotes HNRNPUL1 mRNA stability and accelerates cervical cancer progression. This provides a novel epigenetic aspect in the etiology of cervical cancer. Thus, NAT10 is a newly identified prognostic biomarker for cervical cancer and a promising clinical target for this disease.

## 5. Summary

In conclusion, our study revealed that the ac4C regulator NAT10 is not only positively associated with poor prognosis of cervical cancer, but also promotes tumorigenesis *in vitro* and *in vivo*. We also identified HNRNPUL1 as the direct target of NAT10. The ac4C modification enhances HNRNPUL1 mRNA stability and upregulates its expression in CC.These findings contribute to our understanding of the NAT10-ac4C-HNRNPUL1 for tumorigenesis of CC, meanwhile it might be potential target forcervical cancer therapy.

## Supplementary Material

Supplementary table.
