# Supplementary material for: NAT10-mediated RNA acetylation enhances HNRNPUL1 mRNA stability to contribute cervical cancer progression
Source: Int J Med Sci. 2023 Jun 26;20(8):1079–90. doi: 10.7150/ijms.83828 (PMC10357443; doi:10.7150/ijms.83828)
Supplement: Supplementary file 1 — Supplementary table. [file ijmsv20p1079s1.pdf]

Supplementary Table: Sequences of primers and oligos used in this study

Name	Forward	Reverse
NAT10-sh1	GCAATTGTACACAGTGACTAT	ATAGTCACTGTGTACAATTGC
NAT10-sh2	CGCAAAGTTGTGAAGCTATTT	AAATAGCTTCACAACTTTGCG
hnRNPUL1-sh1	CCGGGATAACAACAACTCCAA	TTGGAGTTGTTGTTATCCCGG
hnRNPUL1-sh2	GCCCGCAAGAAACGCAACTAT	ATAGTTGCGTTTCTTGCGGGC
GAPDH	GATCATCAGCAATGCCTCCT	GAGTCCTTCCACGATACCAA
18S	CGATAACGAACGAGACTCTGGC	CGGACATCTAAGGGCATCACA
SHMT2	CCCTTCTGCAACCTCACGAC	TGAGCTTATAGGGCATAGACTCG
PHGDH	CACGACAGGCTTGCTGAATGA	CTTCCGTAAACACGTCCAGTG
HNRNPUL1	TGAGCACCGAGAGGATAGGAG	GGTGTCAATAGCAACAAGGGT
STC2	GGGTGTGGCGTGTTTGAATG	TTTCCAGCGTTGTGCAGAAAA
STM1	TCAGCCCTCGGTCAAAAGAAT	TTCTCGTGCTCTCGTTTCTCA
RAD23B	CTGAGAGCCAGTTTCAACAACC	ACTGAGGCTGATTCCGTAAAAAT
MCM8	TTTACAGCGATAGCTCTCCTTTG	AGGTGCATCTCTTAGTTCAGTTG
CALM3	GACCATTGACTTCCCGGAGTT	GATGTAGCCATTCCCATCCTTG
